# First Reported Case of Endoscopic Ultrasound-Guided Core Biopsy Yielding Diagnosis of Primary Adrenal Leiomyosarcoma

**DOI:** 10.1155/2018/8196051

**Published:** 2018-10-03

**Authors:** Shaunak R. Mulani, Patrick Stoner, Alexander Schlachterman, Hans K. Ghayee, Li Lu, Anand Gupte

**Affiliations:** ^1^Department of Internal Medicine, University of Florida and Malcom Randall VA Medical Center, Gainesville, FL, USA; ^2^Division of Gastroenterology and Hepatology, Thomas Jefferson University Hospital, Philadelphia, PA, USA; ^3^Division of Endocrinology, University of Florida and Malcom Randall VA Medical Center, Gainesville, FL, USA; ^4^Division of Pathology, University of Florida and Malcom Randall VA Medical Center, Gainesville, FL, USA; ^5^Division of Gastroenterology and Hepatology, University of Florida and Malcom Randall VA Medical Center, Gainesville, FL, USA

## Abstract

Primary adrenal leiomyosarcoma (PAL) is an extremely rare mesenchymal tumor with only a few isolated case reports in the medical literature. Endoscopic ultrasound-guided fine-needle aspiration (EUS-FNA) or endoscopic ultrasound-guided core biopsy (EUS-CB) is a safe, effective modality for sampling lesions in the gastrointestinal tract and adjacent organs, including the adrenal glands. We describe the case of a 50-year-old male presenting with abdominal pain and unintentional weight loss over the course of one year. CT imaging revealed an 8.1 cm heterogeneous left adrenal mass with PET-confirmed metastases to the liver and lung. Pheochromocytoma was ruled out. Adrenal cortical carcinoma was the other critical differential diagnosis. As the patient was not a candidate for surgery, an EUS-FNA and CB were performed on this left adrenal mass revealing a spindle cell neoplasm with extensive necrosis confirming the diagnosis of primary leiomyosarcoma. The patient was treated with chemotherapy with palliative radiation. This case demonstrates the utility of EUS-FNA or CB as modalities that can aid in the diagnosis of adrenal lesions in specific circumstances.

## 1. Introduction

Primary adrenal leiomyosarcoma (PAL) is an extremely rare mesenchymal tumor with less than 35 cases reported in the medical literature [1]. Adrenal masses larger than 5 cm are suspicious of malignancy, and to evaluate resectability, preoperative imaging and screening for distant metastases are necessary. Since PALs grow rapidly, do not produce any adrenal hormonal derangements, and lack characteristic tumor markers or imaging findings, preoperative diagnosis is difficult, and diagnosis is usually made on surgical resection or autopsy [1].

Endoscopic ultrasound-guided fine-needle aspiration (EUS-FNA) or endoscopic ultrasound-guided core biopsy (EUS-CB) is a safe, effective modality for sampling lesions in the gastrointestinal tract and adjacent organs, including the adrenal glands [2]. Several studies have demonstrated EUS-FNA of both the left and right adrenal glands is a safe and accurate method compared to other diagnostic techniques including percutaneous or CT guided adrenal sampling [3]. In centers where EUS expertise is available, it is a minimally invasive alternative to adrenalectomy and percutaneous image-guided biopsy of the adrenal glands with excellent yield for tissue diagnosis [4]. It is also a well-tolerated, outpatient procedure that can help guide the next steps in management rather than relying on biochemical means to solely give a diagnosis of exclusion like PAL. To our knowledge, none of the few reported cases of PAL were diagnosed via EUS-FNA or CB. We, therefore, present the first reported case of PAL diagnosed by EUS-CB.

## 2. Case Presentation

**Figure 1:**
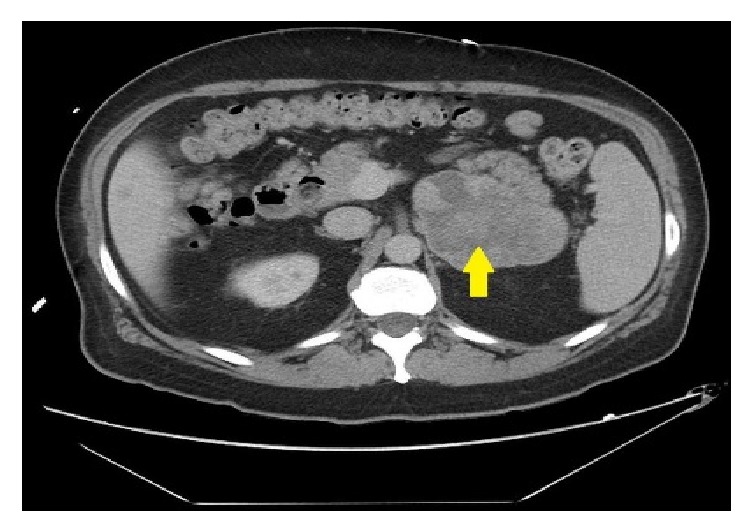
CT of abdomen showing 8.1 cm heterogeneous left adrenal mass.

**Figure 2:**
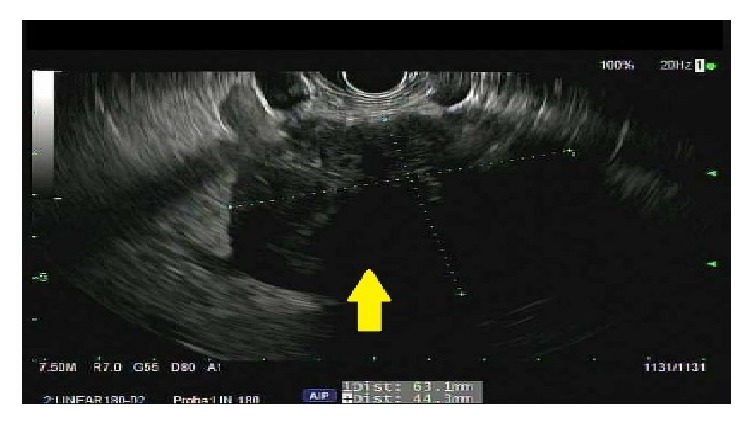
EUS showing irregular, hypoechoic left adrenal mass measuring 63 × 44 mm.

**Figure 3:**
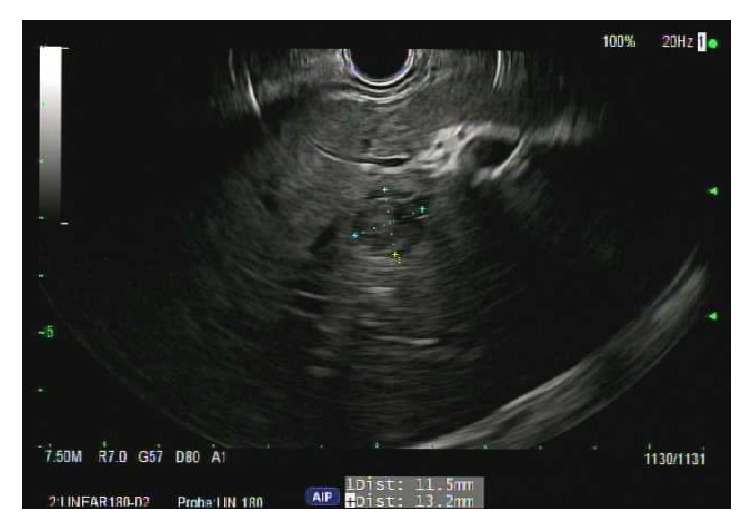
Two hypoechoic round lesions in the left lobe of the liver.

**Figure 4:**
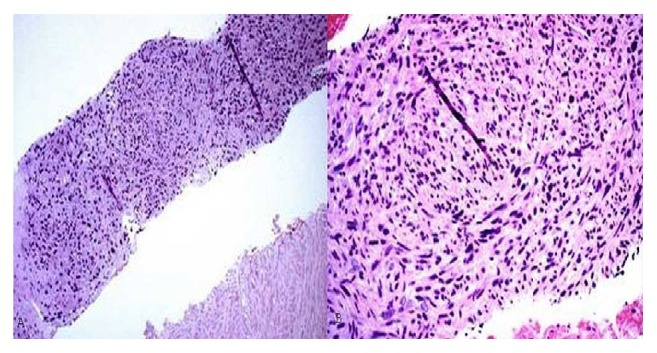
(a) Spindle cell neoplasm with extensive tumor necrosis. (b) High power view showing fascicular growth pattern and cytological pleomorphism.

A 50-year-old male presented with abdominal pain and unintentional weight loss over the course of one year. Physical exam and labs were normal. Computed Tomography (CT) of abdomen showed an 8.1 cm heterogeneous left adrenal mass, several bibasilar lung nodules, and several hypodense liver lesions concerning metastatic disease (Figure 1). Positron Emission Tomography (PET) scan showed FDG avid left adrenal mass and mildly avid lung and liver lesions suggestive of metastatic disease. Pheochromocytoma was ruled out with negative urine metanephrines and catecholamines. There was no clinical evidence of Cushing's Syndrome. However, a nonfunctional adrenal cortical carcinoma (ACC) was the running diagnosis. Since the patient was not a surgical candidate and medical oncology was considering chemotherapy for ACC, EUS-FNA and CB of the left adrenal mass were performed. EUS revealed an irregular, hypoechoic mass, measuring at least 6.3 cm x 4.4 cm in maximal cross-sectional diameter (Figure 2). Two hypoechoic round lesions in the left lobe of the liver were also identified but not sampled (Figure 3). Pathology revealed a spindle cell neoplasm with extensive necrosis (Figure 4). Tumor cells stained positive for desmin, cytokeratin Mak6, WT1, and S-100. Stains were negative for CD34 and chromogranin. Based on these findings, a diagnosis of primary leiomyosarcoma of the left adrenal gland was made. Adriamycin and olaratumab with palliative radiation for pain were then initiated as metastatic disease precluded surgery.

## 3. Discussion

Leiomyosarcomas are soft tissue neoplasms of smooth muscle origin that primarily occur in the myometrium, retroperitoneum, or dermis [1]. PALs, which are extremely rare, originate from the smooth muscle wall of the central adrenal vein and its branches. The first case was reported in 1981 in a 50-year-old patient with a 12 cm lesion arising from the left adrenal vein [5]. Since then, only a handful of additional cases have been reported. As demonstrated in these case reports and in our patient, PALs are associated with delayed diagnosis and poor prognosis. Since they grow rapidly and do not produce hormonal derangements, early diagnosis of PAL is difficult with diagnosis usually made on surgical resection or autopsy [1]. They are typically a diagnosis of exclusion, involving preoperative radiologic imaging and biochemical evaluation to exclude other tumors of the adrenal gland. In the diagnosis of all adrenal lesions, it is important to take into consideration the clinical scenario. All patients should have an adrenal hormonal profile performed first with evaluation of either plasma metanephrines or 24-hour urine catecholamines and metanephrines. Pheochromocytoma must be ruled out prior to adrenal biopsy in order to prevent hypertensive crisis and cardiovascular collapse. Hypercortisolism also needs to be evaluated by utilizing the dexamethasone suppression test. If the patient has a cortisol secreting tumor, then surgery should be performed and not an adrenal biopsy. In addition, nonfunctional ACC must be considered in the differential diagnosis prior to an adrenal biopsy since tumor seeding can take place. Thus the management would be open adrenalectomy and not biopsy. Biopsy can certainly be considered if there is concern for metastatic disease to the adrenal gland or medical oncology needs a tissue diagnosis prior to administering chemotherapy [6].

Since our patient described abdominal pain and weight loss for one year, it was not surprising that metastatic disease was present at the time of diagnosis as PALs are rapidly progressive tumors. Complete surgical resection is the mainstay of treatment for nonmetastatic disease, and the role of chemotherapy or radiation is not well defined [7]. Adriamycin based regimens and radiation for palliation of pain have been reported [1] and are the treatment our patient received. In an open-label phase 1b and randomized phase 2 study, olaratumab plus doxorubicin achieved a highly significant improvement of 11.8 months in median overall survival thus prompting our use of this human antiplatelet-derived growth factor receptor monoclonal antibody in our patient's care [8]. Other modalities of chemotherapy including combinations of vincristine/cyclophosphamide and cisplatin/dacarbazine or external beam radiation have reported limited improvement in patient survival [9].

EUS-FNA is a novel method for diagnosing adrenal lesions in specific circumstances such as in the case described. Before the biopsy was considered, adrenal hormone testing was performed and the possibility of ACC was considered. Since the patient was to undergo chemotherapy, a tissue biopsy was needed; therefore, EUS-FNA was performed. It is a safe procedure with good results, minimal morbidity, and shorter hospital stay [2]. In previous cases, it has had an excellent yield for accurate tissue diagnosis without major complications and only one reported case of minor adrenal hemorrhage [2]. In an EUS approach, only the wall of the stomach or duodenum is traversed to access the adrenal glands unlike in a percutaneous approach. Possible complications after percutaneous biopsies include adrenal hemorrhage, pneumothorax, pancreatitis, adrenal abscess, and needle tract metastases. Thus, compared to a percutaneous approach in which other organs lie in the path of biopsy, EUS-guided biopsy results in lower associated risks [4]. Other advantages of EUS-guided biopsy include needle insertion occurring under real-time ultrasound guidance and FNA or CB which can occur in the same session as a staging EUS. In our case, EUS-FNA yielded a diagnosis of malignancy thereby altering clinical management. Therefore, it is also a useful method to help guide therapy. In our case, the patient tolerated the EUS-CB well without complications and, to our knowledge, was the first case of PAL diagnosed by EUS-FNA or CB.
